# Clinical Reliability of Adjustable Femoral Cortical Suspensory Fixation in Anterior Cruciate Ligament Reconstruction and Correlation of Clinical Outcomes With Demographic and Perioperative Factors

**DOI:** 10.7759/cureus.15345

**Published:** 2021-05-31

**Authors:** Ramesh Kumar, Mukesh Kalra, Ankit Kumar Garg, Ranjeet Choudhary, Nagaraju Venishetty, Shilp Verma, Ankush Kumar

**Affiliations:** 1 Orthopaedics and Trauma, Pushpawati Singhania Research Institute, New Delhi, IND; 2 Orthopaedics, Lady Hardinge Medical College, New Delhi, IND; 3 Orthopaedics, All India Institute of Medical Sciences, Raipur, IND

**Keywords:** adjustable loop cortical femoral suspensory fixation, tightrope, anterior cruciate ligament reconstruction, hamstring grafts, lysholm score, anterior tibial translation

## Abstract

Background and objective

The primary goal of anterior cruciate ligament reconstruction (ACLR) is to protect the initial graft fixation against heavy loads encountered during the rehabilitation phase. The purpose of this study was to evaluate the functional outcomes (Lysholm score) and laxity [anterior tibial translation (ATT), anterior drawer test, Lachman test, and pivot shift test] of ACLR with adjustable-loop femoral cortical suspensory fixation (CSF) and tibial interferences crew fixation.

Methods

This study included 100 patients who underwent primary ACL reconstruction using quadruple hamstring grafts secured with TightRope® (Arthrex Inc, Naples, FL) femoral fixation and an interference screw on the tibial end. Six patients were excluded from the final analysis (four lost to follow-up, one suffered re-injury, and one had septic arthritis). The remaining 94 patients were evaluated for laxity and functional outcomes preoperatively, as well as at one, six, and 12 months postoperatively. Regression analysis was performed to determine the association between outcomes and 11 independent variables. This was designed as a prospective cohort study (level of evidence: II).

Results

The mean age of the participants was 28.46 ± 7.01 years. The median preoperative Lysholm knee score of 49 (mean ± SD: 48.2 ± 5.42) improved to 93 (92.7 ± 2.1) at six months and 98 (97.6 ± 2.1) at the one-year follow-up. The improvement was found to be statistically significant (p<0.01). The median ATT was 10 mm preoperatively, which decreased to 2 mm at one month, remained the same at six months, and rose to 3 mm at the one-year follow-up. ATT was found significantly reduced at one month postoperatively (p<.001) and did not show any significant further changes at subsequent follow-ups (p>0.05). Multiple linear regression revealed that one-year postoperative ATT (Rolimeter, Aircast Europa, Stephanskirchen, Germany) was independent of all demographic and perioperative variables tested.

Conclusion

Quadrupled hamstring graft ACLR with adjustable-loop fixation showed excellent subjective and objective outcomes with no residual laxity or failure of graft over mid-term follow-up. Postoperative laxity was not correlated with graft and tunnel dimensions.

## Introduction

Anterior cruciate ligament (ACL) injuries are most commonly associated with people of young age, especially due to physical activities such as sports, and males are most likely to seek medical attention for them compared to females [1,2]. The treatment approach to ACL tear has undergone several changes in the last four decades. In the early 1990s, surgeons combined the repair of the acute tear with concomitant augmentation of ACL using tendon autografts or synthetic materials [3]. Based on recent evidence, using central one-third bone-patellar tendon-bone and quadrupled hamstring is the most reliable method of autograft for ACL reconstruction (ACLR) [4].

The main objective of ACLR is to ensure a secure initial graft fixation against high loads experienced during early rehabilitation to facilitate tendon-bone healing. Different fixation techniques, such as cortical suspensory fixation (CSF) devices, trans-fixation devices, and interference screws, may be used in ACLR; however, there is no definitive consensus on a standard method as yet. CSF is generally employed for femoral fixation of the graft [2]. A fixed-length loop, a cortical suspension device (EndoButton, Smith & Nephew, Inc., Andover, MA), is commonly used for graft fixation on the femoral end. However, It is associated with the potential drawback of over-drilling the femoral socket to flip the button, resulting in bungee cord effect and windshield wiper effect, thereby contributing to loosening and tunnel widening [4,5]. The fixed-length CSF device is associated with an increased risk of primary ACLR revision [5].

Adjustable-loop length devices, such as TightRope® (Arthrex Inc, Naples, FL) and ZipLoop (Biomet Inc, Warsaw, IN), are second-generation, suspensory fixation devices. The adjustable loop allows the surgeon some flexibility in terms of the length of the femoral socket, avoids the need for cumbersome intraoperative measurements for loop length selection, enables the graft to be re-tensioned even after fixation, and ensures that the tunnel is completely filled with graft [4,6]. We hypothesized that femoral fixation with TightRope would be associated with reduced graft failure and postoperative laxity of the knee. The purpose of this study was to evaluate the functional outcomes (Lysholm score) and laxity [anterior tibial translation (ATT), anterior drawer test, Lachman test, and pivot shift test] of ACLR with adjustable-loop femoral CSF and tibial interferences crew fixation.

## Materials and methods

A prospective cohort study was conducted from December 2015 to February 2017 at a tertiary care center. After obtaining approval from the Institutional Review Board, informed consent was received from 100 patients with an ACL tear in compliance with the 1964 Declaration of Helsinki. The inclusion criteria were as follows: all patients aged more than 19 years who had ACL tear with or without meniscus tear, and without evidence of clinical or radiological degenerative changes in the knee joint. Patients with bony ACL avulsions, ACL tear with other associated ligament injuries, cartilage lesions, intra-articular fractures, ACL tear in the osteoarthritic knee, posterior cruciate ligament tear, bilateral ACL ruptures, and ACLR revision were all excluded.

Surgical technique

All patients underwent standard knee arthroscopy (anteromedial and anterolateral portal); the diagnosis of ACL tear was confirmed, and any additional findings such as meniscus injury and osteochondral lesion were noted. Firstly, meniscal pathology was treated with balancing, debridement, or repairs. Minimal debridement of the ACL stump on both the femoral and tibial sides was performed, as a rule, to preserve the healing capacity of the sheath remnant.

**Figure 1:**
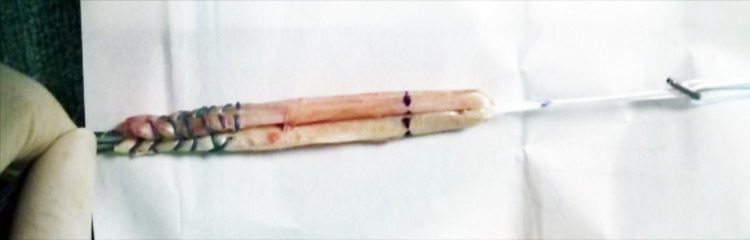
Quadrapled hamstring graft loaded to the TightRope

Hamstring grafts, both semitendinosus and gracilis tendons, were harvested. TightRope fixation was used on the femoral side. The graft was loaded to a tight rope device by symmetrical quadruple folding over the loops (Figure 1).

The femoral tunnel was drilled at a 10 o'clock position for the right or 2 o'clock position for the left knee with the knee flexed 110-120 degrees via the accessory anteromedial portal. Posterior cortical thickness of approximately 1-2 mm was left intact with the help of the posterior offset device. The tibial tunnel was made using the tibial jig set at 55 degrees. Tunnel diameter was determined based on the thickness of the graft. On the tibial side, the graft was fixed with an interference screw after cycling it 20 times. Re-tensioning was done on the femoral side again. The knee was immobilized in full extension with a knee brace. Postoperatively, antibiotics were given for one day, and routine analgesics were prescribed as needed. Suture removal was performed at two weeks postoperatively. All patients underwent institutional ACL rehabilitation procedure starting from postoperative day three as per protocol and continued through the follow-up period of three to six months. Diagnostics and ACLR were performed by a single surgeon with 10 years of experience.

**Figure 2:**
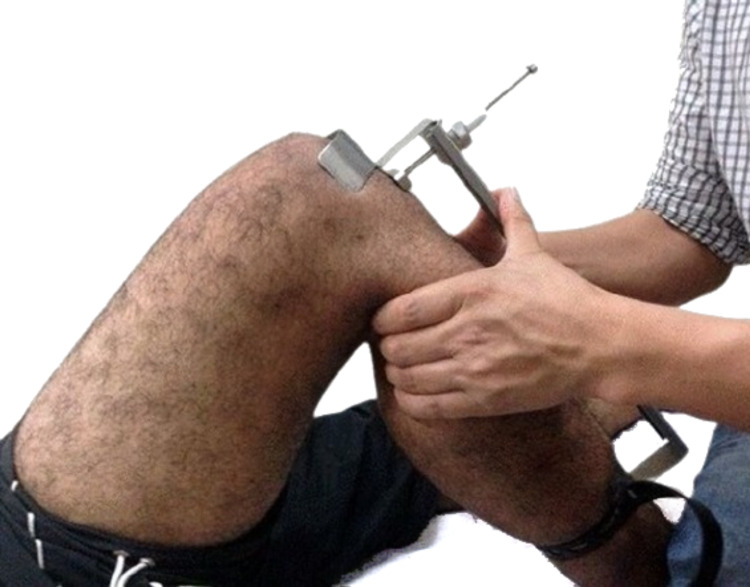
Measurement of anterior tibial translation with Rolimeter

A single, blinded independent observer assessed the clinical outcomes. The Lysholm knee scoring system was used to evaluate the functional improvement. Assessment of the knee laxity was measured by the Lachman test, anterior drawer test, pivot shift test, and ATT (Rolimeter, Aircast Europa, Stephanskirchen, Germany) (Figure 2) [7]. The assessment was performed preoperatively, as well as at one month, six months, and one year postoperatively.

Statistical analysis

Categorical variables were presented as numbers and percentages, and continuous variables were presented as mean ± SD and median. The normality of data was tested with the Kolmogorov-Smirnov test. If the normality was rejected, then the non-parametric test was used. A p-value of <0.05 was considered statistically significant. The data were entered into a Microsoft Excel spreadsheet, and analysis was done using SPSS Statistics version 25.0 (IBM, Armonk, NY).

## Results

A total of 100 patients were initially included in the study. Four patients were lost to follow-up, one had an arthrotomy and debridement for infective septic arthritis, and one had a repeat ACL tear due to a road traffic accident (RTA) at five months postoperatively, and these patients were excluded from the analysis; hence a total of 94 patients were included in the final analysis.

Of the 94 total patients, 60 (63.8%) were male and 34 (36.2%) were female. The mean age of the participant was 28.46 ± 7.01 years; 49 participants (52.1%) had their right knee affected, and 45 (47.9 %) the left knee. The most common mode of injury was RTA, accounting for 44 cases (46.8%) (Table 1).

**Table 1:** Demographic details of the study group RTA: road traffic accident

Variables	Number (n)	Percentage (%)
Age		
<20 years	2	21.27
20-40 years	90	95.74
>40 years	2	21.24
Gender		
Male	60	63.8
Female	34	36.2
Affected limb		
Right	49	52.1
Left	45	47.9
Mode of injury		
RTA	44	46.8
Fall	30	31.9
Sports activity	20	21.3

ACL tear was confirmed in all cases via diagnostic arthroscopy, with tear detected at the mid-substance in 63.8% of patients, femoral attachment in 21.3%, and tibial attachment in 14.9%. No associated injuries were found in 56 patients (60%). Medial meniscus injury was the most common associated injury, occurring in 22 patients (23.3%) (Table 2). The mean time period from injury to surgery was 6.46 ± 5.55 months.

**Table 2:** Diagnostic arthroscopic findings ACL: anterior cruciate ligament

Variables	Number (n)	Percentage (%)
Level of the ACL tear		
Femoral end	20	21.3
Mid-substance	60	63.8
Tibial end	14	14.9
Associated injuries		
Medial meniscus tear	22	23.4%
Lateral meniscus tear	11	11.7%
Both medial and lateral meniscus	5	5.3%
No associated injury	56	59.5%

The mean quadrupled hamstring graft length was 127.0 ± 8.4 mm, and the mean diameter was 7.5 ± 0.81 mm. The mean femoral tunnel length was 39.2 ± 2.5 mm, and the mean tibial tunnel length was 51.6 ± 3.4 mm (Table 3).

**Table 3:** Dimensions of ACL grafts and femoral and tibial tunnels ACL: anterior cruciate ligament; SD: standard deviation

Variables	Mean ± SD (mm)	Range (mm)
ACL graft		
Length	127.0 ± 8.4	110-140
Width	7.5 ± 0.81	6-9
Femoral tunnel		
Length	39.2 ± 2.5	35-48
Width	7.4 ± 0.6	6-9
Tibial tunnel		
Length	51.6 ± 3.4	45-55
Width	7.4 ± 0.6	6-9

**Figure 3:**
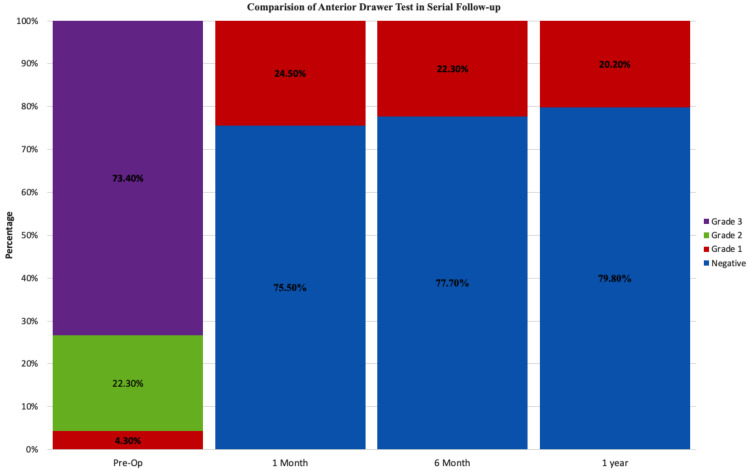
Stacked column plot depicting the improvement in knee laxity as assessed with the anterior drawer test

**Figure 4:**
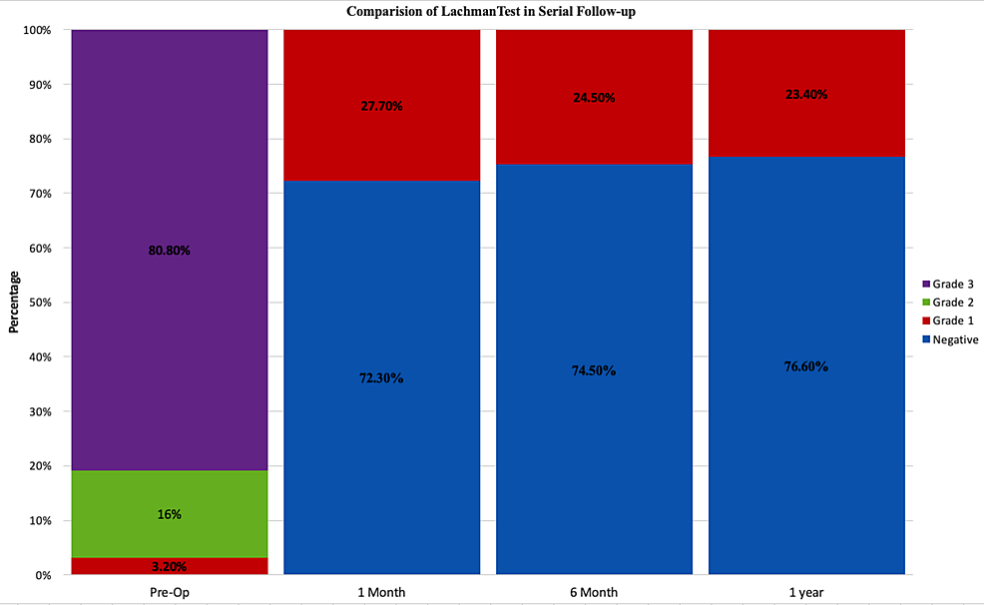
Stacked column plot depicting the improvement in knee laxity as assessed with the Lachman test

The femoral and tibial tunnel diameter was chosen the same as graft diameter. The mean tourniquet time was 52.9 ± 13.2 minutes (range: 34-110 minutes). Anterior drawer test and Lachman test were used to measure anterior laxity. Laxity was classified as negative (0-2 mm), grade 1 (2-5 mm), grade 2 (5-10 mm), and grade 3 (>10 mm) for both these tests [7]. Anterior laxity assessments showed a statistically significant downgrade of laxity at the subsequent follow-ups compared to preoperative levels as per the Wilcoxon signed-rank test (p<0.05). None of the patients showed more than grade 1 laxity one month after surgery. Improvement in laxity was maintained from one month postoperatively till the final follow-up (12 months) and did not show any significant change throughout the entire follow-up period (p>0.05) (Figures 3, 4). Pivot shift tests were used to assess postoperative rotatory laxity (Table 4). The pivot shift test was positive in 76% of cases preoperatively and was negative in all operated knees tested at all subsequent follow-ups.

**Table 4:** Postoperative improvements in subjective clinical parameters (anterior drawer test, Lachman test, and pivot shift test)

Analysis		Preoperative	Postoperative		
			1 month	6 months	12 months
Anterior drawer test					
	Negative, n (%)	-	71 (75.5%)	73 (77.7%)	75 (79.8%)
	Grade 1, n (%)	4 (4.3%)	22 (24.5%)	21 (22.3%)	19 (20.2%)
	Grade 2, n (%)	21 (22.3%)	-	-	-
	Grade 3, n (%)	69 (73.4%)	-	-	-
	P-value		0.001	0.157	0.413
Lachman test					
	Negative, n (%)	-	68 (72.3%)	70 (74.5%)	72 (76.6%)
	Grade 1, n (%)	3 (3.2%)	26 (27.7%)	24 (24.5%)	22 (23.4%)
	Grade 2, n (%)	15 (16%)	-		
	Grade 3, n (%)	76 (80.8%)			
	P-value		0.001	0.157	0.083
Pivot shift test					
	Negative, n (%)	18 (19.1%)		94 (100%)	94 (100%)
	Positive, n (%)	76 (80.9%)			
	P-value			0.001	1.00

When the Kolmogorov-Smirnov test was used to assess data distribution, it was observed that both the Lysholm score and the ATT measurement data had a non-Gaussian distribution, and hence a non-parametric test was used in its analysis.

**Figure 5:**
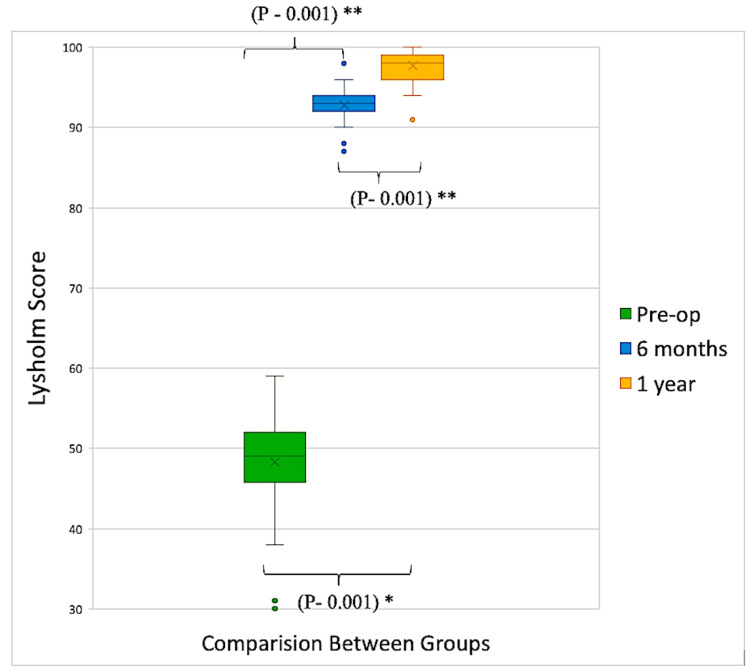
Box-whisker plot indicating improvements in Lysholm scores at sequential follow-ups *Friedman test; **Wilcoxon signed-rank test P-values indicate the significance of overall trends (Friedman test) and differences between consecutive follow-up visits (Wilcoxon signed-rank test). Dots indicate outliers

The median preoperative Lysholm knee score of 49 (mean ± SD: 48.2 ± 5.42) improved to 93 (92.7 ± 2.1) at six months and 98 (97.6 ± 2.1) at the one-year follow-up. The improvement in the Lysholm score at every sequential follow-up was found to be statistically significant after applying the Wilcoxon signed-rank test (p<0.01) (Figure 5) (Table 5).

**Table 5:** Postoperative improvements of laxity (ATT) and Lysholm score ATT: anterior tibial translation

Variables		Median	Mean	Standard deviation	Range	P-value
Anterior tibial translation (Rolimeter)						
Preoperative, mm		10.0	10.7	1.9	7-14	
Postoperative, mm	1 month	2.0	2.4	1.0	1-5	0.01
	6 months	2.0	2.5	0.7	1-5	0.24
	12 months	3.0	2.7	0.7	1-5	0.13
Normal knee (opposite side), mm		3.0	2.6	0.84	1-5	0.74
Lysholm score						
Preoperative		49.0	48.2	5.7	30-59	
Postoperative	6 months	93.0	92.7	2.1	87-98	0.01
	12 months	98.0	97.6	2.1	91-100	0.01

**Figure 6:**
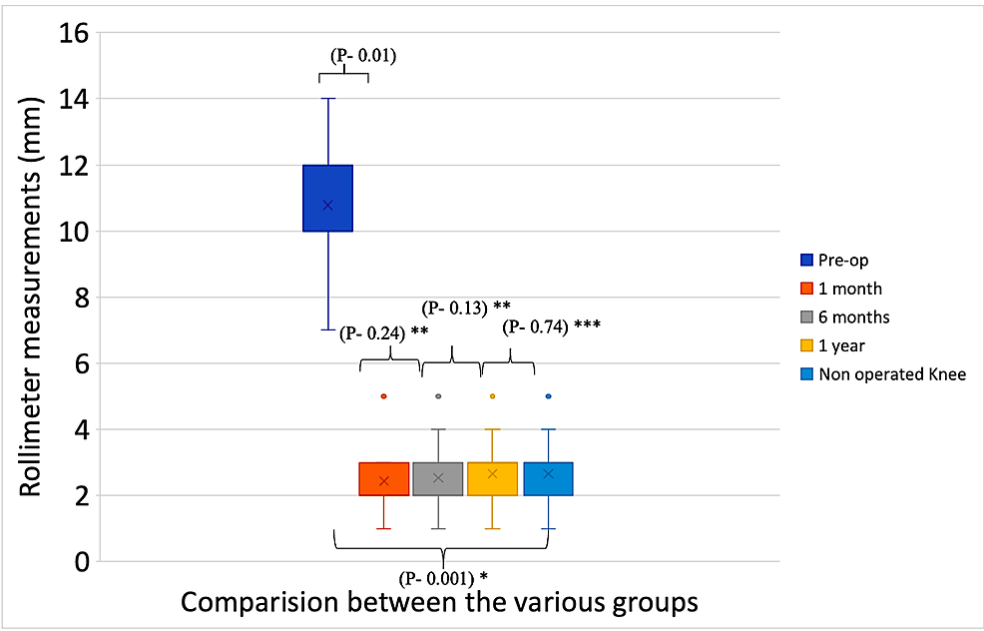
Box-whisker plots indicating improvements in laxity (anterior tibial translation - Rolimeter) at sequential follow-ups *Friedman test; **Wilcoxon signed-rank test; ***Mann-Whitney U test P-values indicate the significance of overall trends (Friedman test), differences between consecutive follow-up visits (Wilcoxon signed-rank test), and differences between the normal knee and anterior laxity of the operated knee at one year (Mann-Whitney U test). Dots indicate outliers

ATT was measured at 90 degrees of knee flexion by Rolimeter. The median ATT was 10 mm preoperatively, which decreased to 2 mm at one month, remained the same at six months, and rose to 3 mm at the one-year follow-up. The anterior laxity was found significantly reduced at the one-month follow-up (p<.001) and did not significantly change on subsequent follow-ups as per Wilcoxon signed-rank test analysis (p>0.05). Postoperatively, none of the patients showed ATT of >5 mm. The mean ATT of the operated knee was comparable to the normal opposite knee at 12 months as per the Mann-Whitney U test (p=0.75) (Table 5) (Figure 6).

Multiple linear regression revealed that ATT at 12 months postoperatively was independent of all demographic and perioperative variables tested (Table 6). In contrast, the Lysholm score tended to increase with the increasing ACL graft length (b=0.085; p=0.008) and decreased with increasing tourniquet time (b=-0.047; p=.005) and femoral tunnel length (b=-0.242; p=.005) (Tables 6, 7).

**Table 6:** Linear regression to identify factors associated with Lysholm score at one year ACL: anterior cruciate ligament

Variables	Regression coefficient (B)	95% confidence interval	P-value
Age	0.068	0.01 to 0.12	0.061
Sex	0.736	-0.18 to 0.16	0.115
Site of the ACL tear	-0.570	-0.06 to 0.02	0.060
Injury-surgery interval	-0.064	-0.14 to 0.01	0.106
Tourniquet time	-0.047	-0.07 to -0.01	0.005
ACL length	0.085	0.02 to 0.14	0.008
ACL width	-0.156	-1.07 to 0.76	0.738
Femoral tunnel length	-0.242	-0.40 to -0.07	0.005
Femoral tunnel width	0.510	-0.123 to 1.148	0.116
Tibial tunnel length	0.122	-0.01 to 0.24	0.058
Tibial tunnel width	-0.085	-1.22 to 1.0	0.882

**Table 7:** Linear regression to identify factors associated with anterior laxity (Rolimeter) at one year ACL: anterior cruciate ligament

Variables	Regression coefficient (B)	95% confidence interval	P-value
Age	0.03	0.01 to 0.05	0.063
Sex	-0.33	-0.68 to 0.01	0.057
Site of the ACL tear	0.23	0.01 to 0.46	0.039
Injury-surgery interval	0.01	-0.01 to 0.40	0.488
Tourniquet time	0.01	-0.01 to 0.02	0.112
ACL length	0.00	-0.02 to 0.02	0.868
ACL width	-0.21	-0.56 to 0.13	0.223
Femoral tunnel width	0.00	-0.06 to 0.06	0.929
Femoral tunnel length	0.140	-0.08 to 0.35	0.208
Tibial tunnel length	-0.01	-0.05 to 0.04	0.872
Tibial tunnel width	0.33	-0.09 to 0.76	0.128

One patient developed a fever, swollen and warm-operated knee, and seropurulent discharge from tibial stitches on the seventh postoperative day. Aspiration of joint fluid revealed Gram-positive *Staphylococcus aureus*, which resulted in a diagnosis of septic arthritis. An open arthrotomy and debridement were performed, antibiotics were administered for three months, and revision ACLR with the contralateral hamstring graft was performed. One patient had a repeat tear of the ACL due to RTA five months after the surgery and underwent revision ACLR. Both of these patients, as mentioned above, were excluded from the final analysis.

## Discussion

With the substantial increase in incidents of sports injury, in addition to the ever-increasing burden of RTAs and domestic injuries, ACL injury is now a common problem encountered by orthopedic surgeons. According to the Norwegian National Knee Ligament Registry, the annual rate of primary ACL reconstruction surgeries is 34 per 100,000 people, with most patients aged 16-39 years [8]. The Swedish Registry has reported that both primary and revision ACLR reviews are more common in men than women [9]. Physically active young males were also the most common demographic profile in our study.

Approximately 90% of patients with ACL tear have sports-related injuries [1,8,9]. Non-contact ACL injuries account for almost 80% of all ACL injuries. ATT and lower extremity valgus are likely vital elements of the injury process when jumping and cutting [1]. The most common cause of ACL tear in this study was RTA, which accounted for 46.8% of the cases. This may be explained by the fact that our region has fewer sports facilities/activities in general and high RTA incidence rates.

Graft fixation devices are a critical element of ACLR since they absorb high stress during the weak link time (the period of tendon-bone healing), which is usually expected to range from six weeks to six months after surgery. Graft fixation devices are categorized based on their mechanism of action: compression (interference screw), expansion (cross pin), or suspension (buttons, Swing Bridge). Due to high ultimate failure strength and construct stiffness, a fixed-loop CSF (EndoButton) is widely used for femoral end fixation of grafts [10,11]. Bungee cord and windshield wiper effects have been associated with tunnel widening and reduced bone-graft tissue incorporation [12,13]. Over-drilling of the femoral tunnel is needed to allow it to flip at the femoral cortex, leaving the scope of the socket unfilled by the graft (attic), and over-drilling is often not possible due to the small anatomical femoral condyle [13]. The greater height of the attic (void above the graft in the femoral socket) of the femoral tunnel is associated with increased graft movement in the tunnel and increased femoral tunnel widening [14].

An adjustable-loop CSF device (TightRope) has been designed to address the concerns mentioned above regarding fixed-loop devices. Graft tension can be lost during the insertion of the tibial interference screw fixation. Unlike a fixed-loop device, an adjustable-loop device could address it by re-tensioning the graft after definitive tibial fixation [6]. This study was designed to assess the subjective and objective outcomes of ACLR with respect to TightRope femoral fixation and tibial biodegradable interference screw.

Theoretically, cyclic loading of the knee could cause loop slippage and elongation of adjustable CSF during postoperative rehabilitation. TightRope has demonstrated clinically significant increased loop lengthening during prolonged cyclic loading in the laboratory when compared to EndoButton. However, the ultimate load of both devices exceeds the usual physiology force of the recovery phase [15]. Though biomechanical analysis is beneficial, the study's laboratory-based design limits its clinical applicability and replicability because load transfer in vivo is seldom purely linear along the graft's direction, as it is in laboratory studies, and loading force is usually below the elongation threshold [10,11].

In this study, subjective assessments of anterior laxity showed a statistically significant downgrade of laxity grade at subsequent follow-ups (p<0.05), which was maintained from one month postoperatively till the last follow-up. None of the patients showed more than grade 1 laxity after the surgery. Similarly, an objective assessment of anterior laxity was performed by measuring ATT with Rolimeter. Rolimeter measurements are a reliable method for measuring anterior knee laxity, including in the acute phase of knee injuries [16]. Median ATT was found to be decreased significantly from 10 mm preoperatively to 2 mm at one month (p<.05), remained the same at six months, and rose to 3 mm at the 12-month follow-up (p>.05). The mean ATT of the operated knee was comparable to the normal opposite knee at 12 months (p=0.75). There was no clinical loosening or laxity until the last follow-up, as reported by various biomechanical studies [10,11,15].

The Lysholm score, a patient-administered instrument used to assess ACL and associated knee injury, consists of psychometric parameters such as reliability, consistency, floor and ceiling effects, validity, and responsiveness [17]. Lysholm score at all sequential follow-up among our patients was statistically significant after applying the Wilcoxon signed-rank test (p<0.01).

According to regression analysis, one-year postoperative laxity (ATT: Rolimeter) was unaffected by demographic or preoperative parameters. In terms of biomechanics, ultimate load and stretch resistance are proportional to graft diameter [18]. However, clinically, a thinner graft does not increase residual laxity or revision rate [19]. Anatomic reconstruction is a more important factor than graft size; increased graft size did not enhance knee stability during reconstruction [18].

However, a shorter duration of tourniquet inflation was associated with a higher postoperative Lysholm score. Larger ACL graft length and shorter femoral tunnel length are associated with a high Lysholm score postoperatively. Given the lack of correlation between postoperative laxity and perioperative factors (graft and tunnel dimension), this novel association with the Lysholm score warrants further investigation.

TightRope button fixation has been reportedly associated with various technical difficulties such as button jamming in the femoral guide pin hole, non-flipping even after passing through the lateral femoral, and pull-out through the skin laterally or flipping within the substance of the vastus lateralis, which might lead to the failure of reconstruction [20]. These complications can be avoided by visualizing the TightRope button in the femoral socket with the arthroscope. We did not encounter such difficulties or any early failure of reconstruction.

Limitations

This study has some limitations. The lack of a control group is the primary drawback of this analysis, as it rendered a direct comparison with other suspension devices impossible. Secondly, the follow-up time was comparatively short, with the maximum follow-up period being one year. However, according to the literature, a considerable proportion of graft defects occur during the early postoperative period [21]. Finally, no radiographic analysis of tunnel widening was performed at follow-ups. So it is unknown whether the adjustable-loop fixation improves upon tunnel widening complication associated with a fixed-loop device. Despite these constraints, the current study provides a robust subjective and objective analysis of the outcomes of ACLR using an adjustable CSF system, which has been shown to be promising in the mid-term. Further studies with larger sample sizes and longer follow-ups are required to draw definitive conclusions and design treatment guidelines.

## Conclusions

Based on our findings, an adjustable-loop CSF device on the femoral side further tightens the graft if needed even after the tibial end fixation is done. The quadrupled hamstring graft ACLR with adjustable-loop fixation has shown excellent outcomes with no residual laxity or failure of the graft, based on analysis with mid-term follow-ups. Postoperative laxity was not correlated with graft and tunnel dimensions. However, more comparative studies with larger sample sizes are required to gain more insight into the subject.
